# Frontalis suspension surgery to treat patients with essential blepharospasm and apraxia of eyelid opening-technique and results

**DOI:** 10.1186/1746-160X-10-44

**Published:** 2014-10-22

**Authors:** Chrisanthi Karapantzou, Dirk Dressler, Saskia Rohrbach, Rainer Laskawi

**Affiliations:** ENT-Department, University of Göttingen Medical Center, Göttingen, Germany; Department of Neurology, University of Hannover Medical Center, Hannover, Germany; Department of Audiology and Phoniatrics, University of Berlin Medical Center, Berlin, Germany; HNO-Klinik Universitätsmedizin Göttingen, Robert-Koch-Str. 40, 37075 Göttingen, Germany

**Keywords:** Blepharospasm, Apraxia of eye lid opening, Frontalis suspension surgery

## Abstract

**Introduction:**

We describe the results of 15 patients suffering from essential blepharospasm with apraxia of eyelid opening who underwent frontalis suspension surgery.

**Material and methods:**

Patients with apraxia of eyelid opening and unresponsive to botulinum toxin injections were studied. Bilateral frontalis suspension surgery was performed (sling operation) using polytetrafluoroethylene (Gore-Tex®) sutures. The patients reported the *degree of improvement* using a subjective rating scale to evaluate the benefit of the operation at two times after surgery (0-10 days and 180-360 days).

**Results:**

The patients reported a high degree of subjective improvement. In the early postoperative period (0-10 days) the mean degree of subjective improvement was 74.6% (standard deviation (SD) 26.4%). At 180-360 days after surgery the mean improvement was 70.0% (SD 26.7%). Small hematomas of the upper lid occurred postoperatively in all patients. Other complications were suture extrusions (9.1%), suture granulomas (6.1%), lacrimation (5.0%) and local infections (7.5%). Postoperatively, all patients needed additional botulinum toxin injections for optimal outcome.

**Conclusion:**

Frontalis suspension surgery is a minimally invasive and effective treatment option for apraxia of eyelid opening in patients with essential blepharospasm unresponsive to botulinum toxin injections alone.

## Introduction

**Figure 1:**
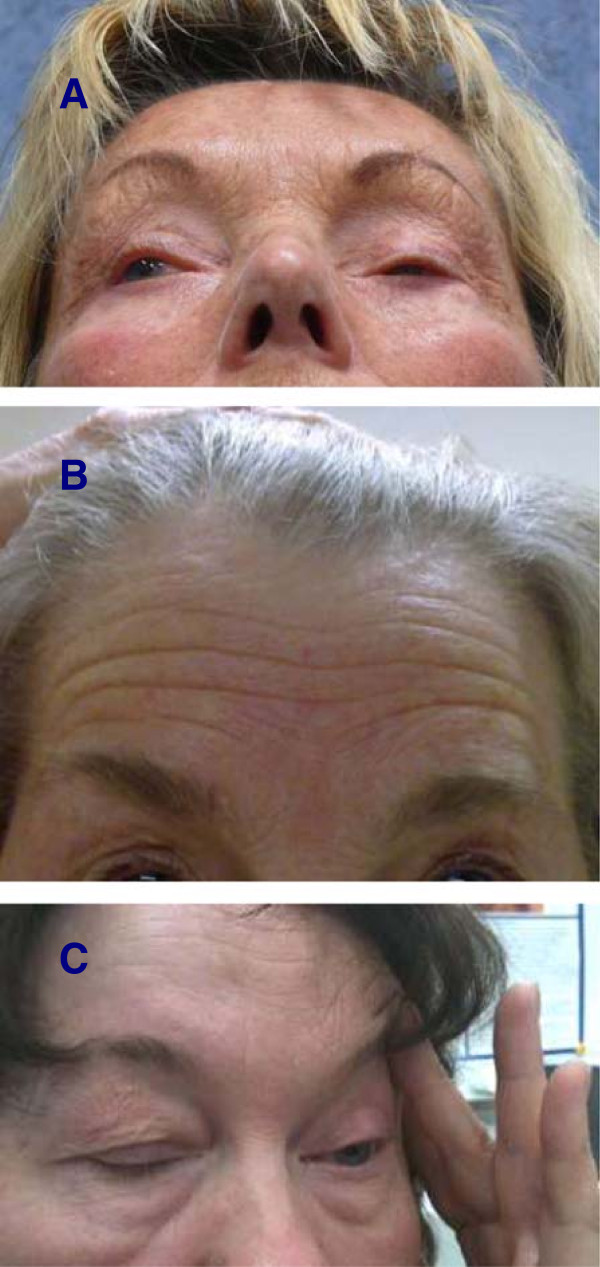
**Clinical presentation and compensatory maneuvers in patients with blepharospasm and apraxia of eyelid opening. A**: Patient with apraxia of eyelid opening. Retroflexion of the head. **B**: Innervation of the frontalis muscle as a compensatory maneuver to increase eye opening. **C**: Lifting the upper lid with a finger to open the eye.

The treatment of essential blepharospasm with botulinum toxin (BoNT) is a well-established method [1, 2]. However, some patients suffer from a special form of blepharospasm that renders them unable to open their eyes adequately despite local BoNT treatment. This phenomenon is due to the inability to raise the eyelid (see Figure 1). This is referred to in the literature as “apraxia of eyelid opening”, “levator inhibition blepharospasm” or “dystonic eyelid opening disorder” [3–6]. It leads to a compensatory constrained posture such as retroflexion of the head to enable the patient to see through the narrow palpebral fissure. The patients frequently exhibit photophobia, and perform certain compensatory movements to keep the palpebral fissure as wide as possible. Many patients increase vertical traction by contracting the frontalis muscle to widen the opening of the palpebral fissure. Since BoNT treatment alone will not provide any significant improvement of the eye opening, a number of more or less invasive surgical procedures have been introduced [3–5, 7, 8]. In our patients with this condition we perform frontalis suspension surgery (“sling operation”) based on the technique of Roggenkämper and Nüssgens [3, 4]. In this report we describe this surgical procedure and present our results of the operation as well as the assessments of the patients with regard to the success of the treatment and to their quality of life.

### Patients and methods

#### General information

This retrospective analysis, which had the approval of our institutional ethics review board (University of Göttingen Medical Center), encompasses 15 patients, nine women and six men, who underwent bilateral frontalis suspension surgery (surgical details given below) for insufficiency of the lid retractors. All patients had had preoperative BoNT therapy, which by itself had not enabled adequate eyelid opening. A total of 40 eyelid operations, including revisions (number see below) were performed. Seven patients were operated on in one session and eight in a two-stage procedure. A total of ten surgical revisions of five eyelids were necessary in three patients. Thirty-six operations (90%) were performed in general anesthesia with intubation. The patients participated voluntarily in the postoperative interviews (see below). Photographs of patients are published with their written consent.

#### Surgical technique

**Figure 2:**
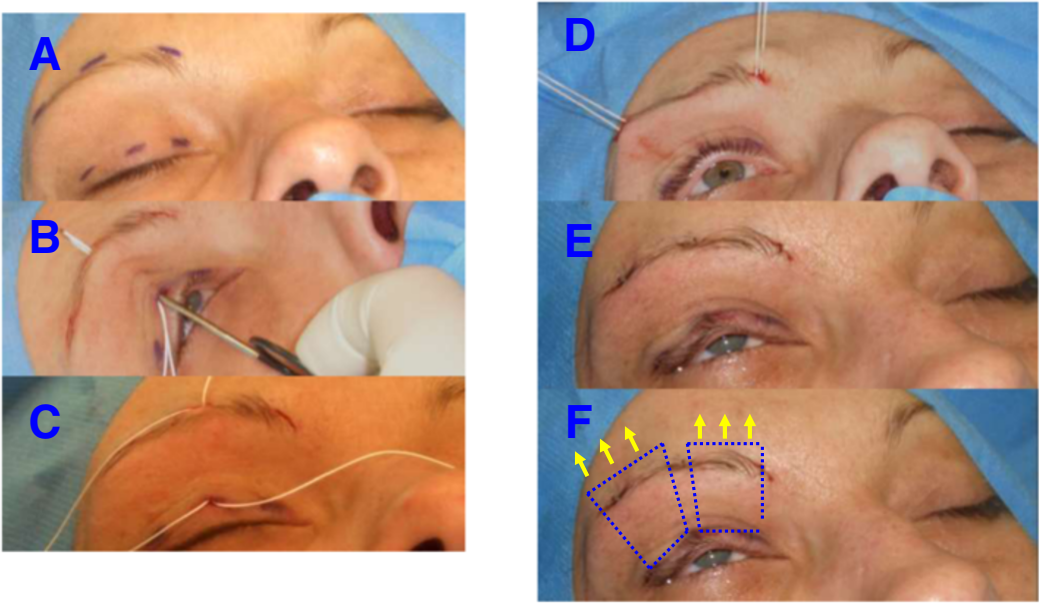
**Steps in frontalis suspension operation. A**: Typical incisions in the upper eyelid and above the eyebrow. **B**: Subcutaneous insertion of polytetrafluoroethylene (Gore-Tex®) sutures from the edge of the upper eyelid to the caudal portion of the frontalis muscle. **C**: The two sutures are positioned to form the lateral and medial two loops (squares) of rectangular shape**. D**: Sutures brought out laterally and medially before tying the knots. **E**: Final status with desired slight opening of the eyelid after tying and burying the knots and stitching the skin incisions. The final step is to apply eye ointment and a special dressing that allows both eyes to be opened immediately after surgery. **F**: The subcutaneous position of the polytetrafluoroethylene sutures is illustrated in an idealized manner. The arrows indicate the direction of force of the frontalis muscle. By suspending the upper eyelid from the caudal frontalis muscle, the upper eyelid can be actively raised by the patient.

All primary operations (based on [3, 4], n = 30) were performed in general anesthesia. The eyebrows and the eyelids were infiltrated subcutaneously with a local anesthetic containing epinephrine (1% Ultracain®) to reduce local bleeding. After disinfecting the skin (Braunol®), and sketching the incision lines above the upper edge of the lid and slightly above the eyebrow (Figure 2) we made three skin incisions and locally undermined the skin so that the polytetrafluoroethylene (PTFE, Gore-Tex®) sutures and knots used for the suspension could be buried as deeply as possible (Figure 2). A lid plate was inserted under the eyelid during the incision to protect the eye. Depending on the thickness of the eyelid, a size 3-0 (87%) or 4-0 (13%) suture was passed through the medial caudal incision in the upper border of the eyelid cephalad to the medial incision just above the eyebrow. A needle was used to pass the suture subcutaneously (see Figure 2). On retraction, the needle was used to pull a second subcutaneous PTFE suture caudally through the medial incisions (Figure 2). Starting from the medial incision, two loops (squares) of rectangular shape were then formed with subcutaneous PTFE sutures (medial, lateral, Figure 2). In order to form an adequate angle, the sutures were led out of the other incisions (lateral, medial) and then reinserted subcutaneously in the desired direction. Finally, the ends of each suture were brought out through the medial upper incision for the first square and out of the lateral upper incision for the second square to be tied (Figure 2). The surgical site was disinfected after each step in placing the sutures. The skin incisions, except for the lateral and medial upper incisions above the eyebrow, were then closed (Figure 2). The position of the eyelid was assessed and adjusted to give a slight overcorrection with a discrete 2-3 mm wide palpebral fissure. The protruding sutures were knotted and buried subcutaneously. The lateral and medial upper incisions were then closed with skin sutures. We used thin, absorbable sutures (Vicryl® 7-0) for skin closure to avoid having to remove them later (Figure 2). Ointment (Polyspectran®) was applied to the eyes, and the forehead and upper eyelid were covered with a sterile dressing.

#### Subjective evaluation of treatment success

Postoperative “satisfaction” with the operation was assessed individually for each eye see [3, 4]. The left and right side were evaluated separately because the patients might possibly register a difference, e.g. eye-opening not equally good or bad on both sides, or because the eyes were operated on separate occasions. The patients were requested to give a subjective evaluation of the success of treatment at two different time windows. Success was scored on a scale of zero to 100 percent. Zero percent was the “lack of any improvement after the operation”, whereas 100% was “optimal treatment results and resolution of all complaints after the operation”. The assessment periods were between day 1 to 10 (early postoperative period) and between day 180 to 365 (late postoperative period) after surgery. The percentage values for the left and the right eye were added for each patient, and the average and standard deviation (SD) were calculated. The average group value of each time period was obtained by calculating the average and standard deviation of the individual averages during that period.

Thirteen patients were able to assess the results in both time periods, while two patients were only able to assess the first period, since the required interval for the second period had not elapsed since the operation.

## Results

### General information

The average interval between the first manifestation of the disorder and the diagnosis was 2.9 years (SD 1.9 years; range 0 to 7.0 years). The average interval between initial diagnosis and begin of BoNT therapy was 0.73 years (SD 1.78; range 0 to 7.0 years).

The average interval between starting local BoNT treatment and the first frontalis suspension operation was 2.8 years (SD 1.7 years; range 1.0 to 6.0 years).

### Surgical technique and complication rate

**Figure 3:**
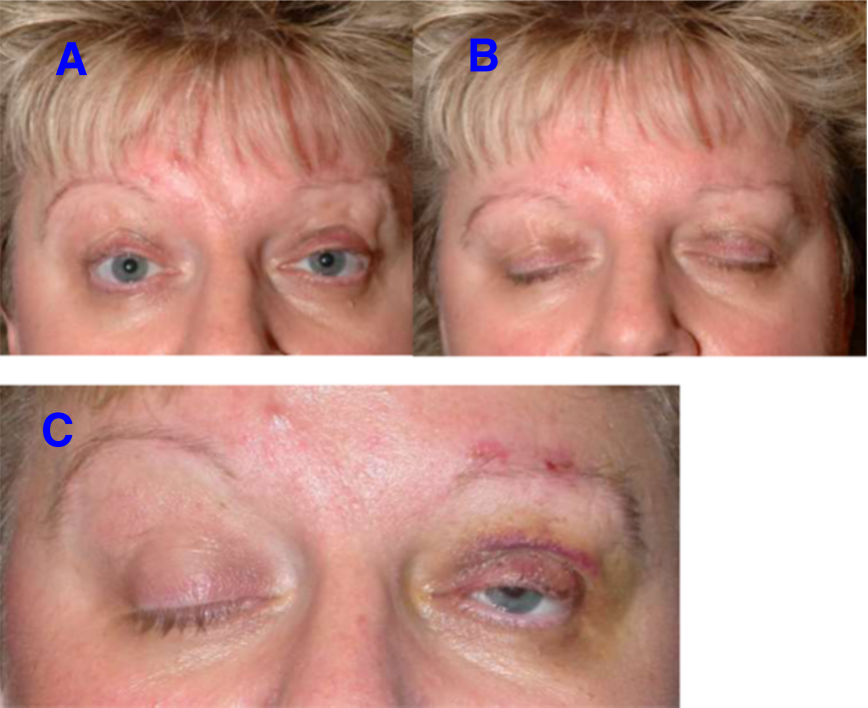
**Patient after bilateral frontalis suspension surgery.** The eyes can be opened **(A)** and closed **(B)** without difficulty. The patient’s BoNT treatment was continued. Insert **C** shows the situation immediately after the unilateral operation of the left eye, identifiable by the small hematoma. One can clearly see that the apraxia persists in the non-operated right eye and that eye opening is not possible in spite of innervation of the frontalis muscle. The position of the eyebrow is higher and the upper eyelid is completely closed. After the suspension surgery, the left, operated eye is already partly opened with only a low-intensity innervation. This is consistent with a lateralized control of the apraxia.

All patients initially reported an improvement of their symptoms (see example in Figure 3). No major complications occurred during surgery or in the immediate postoperative period. The typical and unavoidable postoperative local hematomas and discrete edema of the upper lid were seen in all patients. The relevant postoperative events are summarized in Table 1. During the study period, including revision operations, a total of 66 PTFE sutures or loops (squares) of rectangular shape were placed in the fifteen patients. Six sutures had to be removed during this period because of local tissue reaction giving an extrusion rate of 9.1% (see Table 1). The interval between surgery and reaction to the suture or to revision surgery was two weeks to five months (mean 2.6 months; SD 1.98 months).

**Table 1:** **Type and incidence of postoperative complications**

Postoperative complications	Frequency
Suture extrusion	6 of 66 sutures (9.1%)
Suture granuloma	4 of 66 sutures (6.1%)
Lacrimation	2 of 40 operated eyelids (5.0%)
Infection	3 of 40 operated eyelids (7.5%)

BoNT treatment was continued in all patients, since the effect of the operation alone, although it greatly improved the situation, was not sufficient for an optimal result.

### Individual observations

Several interesting details became apparent when the results of the individual patients who had undergone the frontalis suspension procedure were analyzed. These are presented in two short case reports.

#### Case 1

A 56-year old female patient underwent bilateral two-stage frontalis suspension surgery for typical blepharospasm with apraxia of eyelid opening (Figure 3). After the first operation we noticed that the levator inhibition effect appeared to differ between the two sides (Figure 3). When the patient attempted to raise her eyebrows we observed an increased innervation of the frontalis muscle on the non-operated side but without a resulting adequate lift of the eyelid. A markedly slighter innervation of the frontalis muscle of the treated side gave a much wider opening of the eyelid (Figure 3).

#### Case 2

A 73-year old male patient with multifocal dystonia presented with blepharospasm and apraxia of eyelid opening. Following successful frontalis suspension surgery the PTFE sutures were expulsed on both sides after two months (right side), respectively five months (left side). Despite repeated revision operations with the aim of burying the knots the sutures eventually had to be removed. Despite this the patient continued to be able to open his eyes. The effect persisted for seven months (right eye), respectively 11 months (left eye).

### Subjective rating of success of treatment

All of the fifteen patients reported an initial postoperative improvement of eye opening.

The average early postoperative (0 to 10 days) subjective rating of therapeutic success was 74.6% (SD 26.4%; range 20 to 100%). The average rating at the later period (180 to 365 days) was 70.0% (SD 26.7%; range 0 to 100%).

## Discussion

One main approach in the treatment of essential blepharospasm is to reduce muscle mass in the cranial portion of the orbicularis oculi muscle, often together with parts of the procerus and the corrugator supercilii muscles [7]. These procedures can be combined with suspension operations [8]. They are considerably more invasive than suspension operations alone, as performed in our own and other institutions [3–5].

The polytetrafluoroethylene (PTFE, Gore-Tex®) sutures gave satisfactory functional results. However, it is well known that PTFE sutures do not always integrate well into the tissue bed, and they can cause problems with tissue reactions and extrusion [9–11]. The extrusion rate of 9.1% and the 6.1% incidence of granulomas in our patients are similar to those described by other authors [5]. It should be mentioned that whenever a suture that was introduced using our method is extruded, a new one can be inserted without any problems after a waiting period of only a few weeks. In addition, if the traction effect of the suture decreases, it is possible to uncover the knot and “reposition” the upper eyelid.

Fascia lata gives very good functional results compared to other materials [12, 13], but we prefer polytetrafluoroethylene because this is a minimally invasive option to elevate the upper lid in patients with blepharospam with apraxia of eye lid opening. The removal of fascia lata is more invasive, an additional skin incision is needed in another body region (thigh), and the results do not differ much from the use of polytetrafluoroethylene in this connection [12, 13].

Our observations in individual patients were interesting. In our surgically treated patients, we did not observe the known effect that a unilateral BoNT injection can cause bilateral improvement of blepharospasm. The observed phenomenon of a lateralized change in the ability to raise the eyelid is an argument for a lateralized “organization” of the apraxia of eyelid opening. Another observation was that adequate eye opening could persist even after a suture had to be removed. This phenomenon has not been described previously in the literature. In the case described here, a new suture was implanted in a different position after an appropriate waiting period. The observed phenomenon could be due to the development of filiform scar tissue that, for a limited time, could still give traction on the upper eyelid when the frontalis muscle contracted. This could also be an effect of the remaining sutures if not all had been removed. In this case, the remaining sutures maintain a connection between the upper eyelid and the caudal part of the frontalis muscle.

All patients had preoperative BoNT therapy, but this alone was unable to allow sufficient elevation of the eyelids. The patients rated the treatment results as an improvement, which underscores the positive effect of the operation. It was not possible to discontinue BoNT treatment in any of the patients, since alleviation of the orbicularis oculi muscles spasms appears to be in varying degrees an essential component for optimal results. It is relevant in this context that the BoNT injections during postoperative treatment should not be placed in the caudal portion of the frontalis muscle. This is very important since the strength of the frontalis muscle and the ability to raise the upper eyelid would be reduced if this recommendation were to be ignored. The study by Grivet et al. [14] is of interest in this context. The authors reported that the immediate postoperative improvement of the “Functional Disability Scores” (FDS) was significantly greater in those patients with blepharospasm whose BoNT treatment had been discontinued postoperatively compared to those whose treatment had not been interrupted. But the FDS of the patients with postoperatively continued BoNT injections improved later to the level of the patients without postoperative BoNT treatment. In their analysis, however, the authors did not stratify their patients by subtypes or by surgical method. If such a total absence of spasms of the orbicularis oculi muscle is basically relevant in patients with apraxia of eyelid opening remains undecided. Our data, which show that continuing postoperative BoNT treatment was necessary in all patients, argue against that assumption.

The average subjective improvement ratings of our patients were between 75% and 70%, which is a relatively high level see [3–5]. Roggenkämper and Nüssgens [3, 4] observed a stable effect for their patient population as a whole, a result that is similar to the analysis of the follow-up period of our study. But this retrospective study does have some limitations. The results would have been more robust had the improvement of eye opening described by the patients been confirmed by objective measurements. A validated questionnaire and score for the patients’ self-assessment with focus on the degree of eye opening would also have been useful. A further study with a greater number of patients would be useful more detailed insights into this rare and complex disease and determine the therapy options.

In summary one can conclude that frontalis suspension surgery using polytetrafluoroethylene (Gore-Tex®) sutures is a minimally invasive and effective option for the treatment of apraxia of eyelid opening in patients with essential blepharospasm. The quality of daily life and general patient satisfaction is improved. The *combination* with *botulinum toxin* injections seems to be of advantage for an optimal treatment outcome.
